# Advances in biosynthesis of chiral amide herbicides and the key enzymes: dimethenamid-P and S-metolachlor as case studies

**DOI:** 10.1186/s40643-025-00851-2

**Published:** 2025-03-10

**Authors:** Feng Cheng, Yi-Ling Zhou, Dan-Chen Yang, Ding-Yi Zhao, Ya-Ping Xue, Yu-Guo Zheng

**Affiliations:** 1 Zhejiang Laboratory of Bioorganic Synthesis, Zhejiang University of Technology, Hangzhou, 310014 People’s Republic of China; 2 The National and Local Joint Engineering Research Center for Biomanufacturing of Chiral Chemicals, Zhejiang University of Technology, Hangzhou, 310014 People’s Republic of China; 3 Engineering Research Center of Bioconversion and Biopurification of the Ministry of Education, Zhejiang University of Technology, Hangzhou, 310014 People’s Republic of China

**Keywords:** Chiral amide herbicides, Biosynthesis, S-metochlor, Dimethenamid-P, (S)-1-methoxy-2-propylamine

## Abstract

Chiral amide herbicides represent a significant class of agrochemicals, widely used for effective weed control. Prominent examples include *S*-metolachlor and dimethenamid-P, both of which share the intermediate (S)-1-methoxy-2-propylamine, a key structural component in their synthesis. Developing green and sustainable methods for producing this intermediate is crucial for enhancing the environmental and economic feasibility of herbicide manufacturing. Biosynthesis, with its advantages in sustainability and efficiency, has emerged as a pivotal approach in pesticide production. This review explores the classification and current development status of chiral amide herbicides, including their varieties and applications in the agricultural market. It outlines the synthesis pathways for S-metolachlor and dimethenamid-P, covering both chemical and biosynthetic routes. The review also highlights the functional properties of the key enzymes involved in the biosynthesis of (S)-1-methoxy-2-propylamine, focusing on the potential for enzyme engineering and creation to optimize these pathways. The challenges and future development directions for amide herbicides are discussed, with an emphasis on overcoming synthetic and ecological barriers.

**Graphical Abstract:**

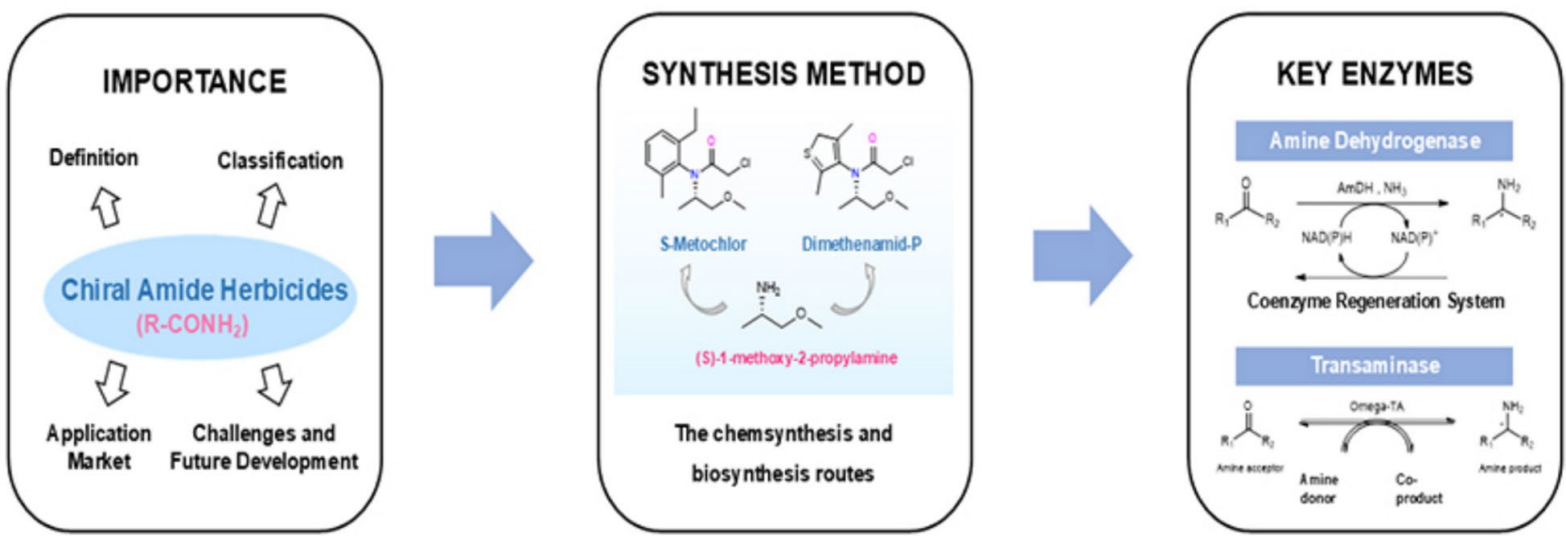

## Introduction

Herbicides are the most widely used pesticides, significantly boosting global agricultural yields. Amide herbicides rank fourth in the global market, following amino acid and sulfonylurea herbicides (Zhang 2011a). These herbicides, effective against grass weeds, are used in crops like soybeans, corn, peanuts, cotton, and potatoes. Their active ingredients, phenoxycarboxylic acid compounds, inhibit plant growth by disrupting metabolism (Wu et al. 2023). Common examples include acetochlor, metolachlor, butachlor, alachlor, and propisochlor, with acetochlor, butachlor, and metolachlor being the most widely used.

Notably, many amide herbicides exhibit optical isomerism. More than 40% of commonly used pesticides have chiral structures (Cui et al. 2019), and their enantiomers can exhibit vastly different pharmacokinetics, metabolism, and toxicological properties. The significance of chirality was first recognized following the thalidomide tragedy in the 1960s, where the S-enantiomer of the drug caused severe birth defects in over 10,000 children (Zhang et al. 2019). However, due to cost limitations and technological challenges, most commercial pesticides are still marketed as racemic mixtures (Meng et al. 2022; Drăghici et al. 2013), with only a limited number of enantiomerically pure pesticides being successfully developed and applied (Vashistha et al. 2024). For amide herbicides, metolachlor and alachlor stand out as optically active examples with significantly enhanced activity in their chiral forms. Metolachlor was patented in 1982, with its enantiomerically enriched form patented in 1992, achieving a nearly twofold reduction in application rates (750–1500 g/m^2^ for the racemic form vs. 400–820 g/m^2^ for the chiral form). Similarly, alachlor was patented in 1972, with its enantiomerically enriched form patented in 1981, also achieving a nearly twofold reduction in application rates. Today, chiral alachlor accounts for more than 70% of the alachlor market worldwide.

The production of herbicides poses significant toxicity and environmental challenges, particularly regarding water pollution and endocrine disruption. During the production of optically active forms, the inactive enantiomers often accumulate in the environment, leading to selective enrichment and migration (Zhou et al. 2021), thereby causing ecological harm. Amide herbicides such as butachlor, acetochlor, and metolachlor have been banned in the European Union and are strictly regulated in the United States. Another significant attention is given to the “three wastes” (wastewater, waste gas, and solid waste) in pesticide production. In 2003, the Chinese Ministry of Environmental Protection established wastewater discharge standards for pesticide manufacturing and imposed limits on impurities in amide herbicides. It is imperative to address the challenges associated with the “three wastes,” impurities, and chiral separation in amide herbicides. Developing efficient, green, and environmentally friendly methods for producing high-activity chiral enantiomers and comprehensively evaluating their environmental behavior, bioactivity, and ecological toxicity will be critical for ensuring the sustainable development of these herbicides (Meng et al. 2022).

**Fig. 1:**
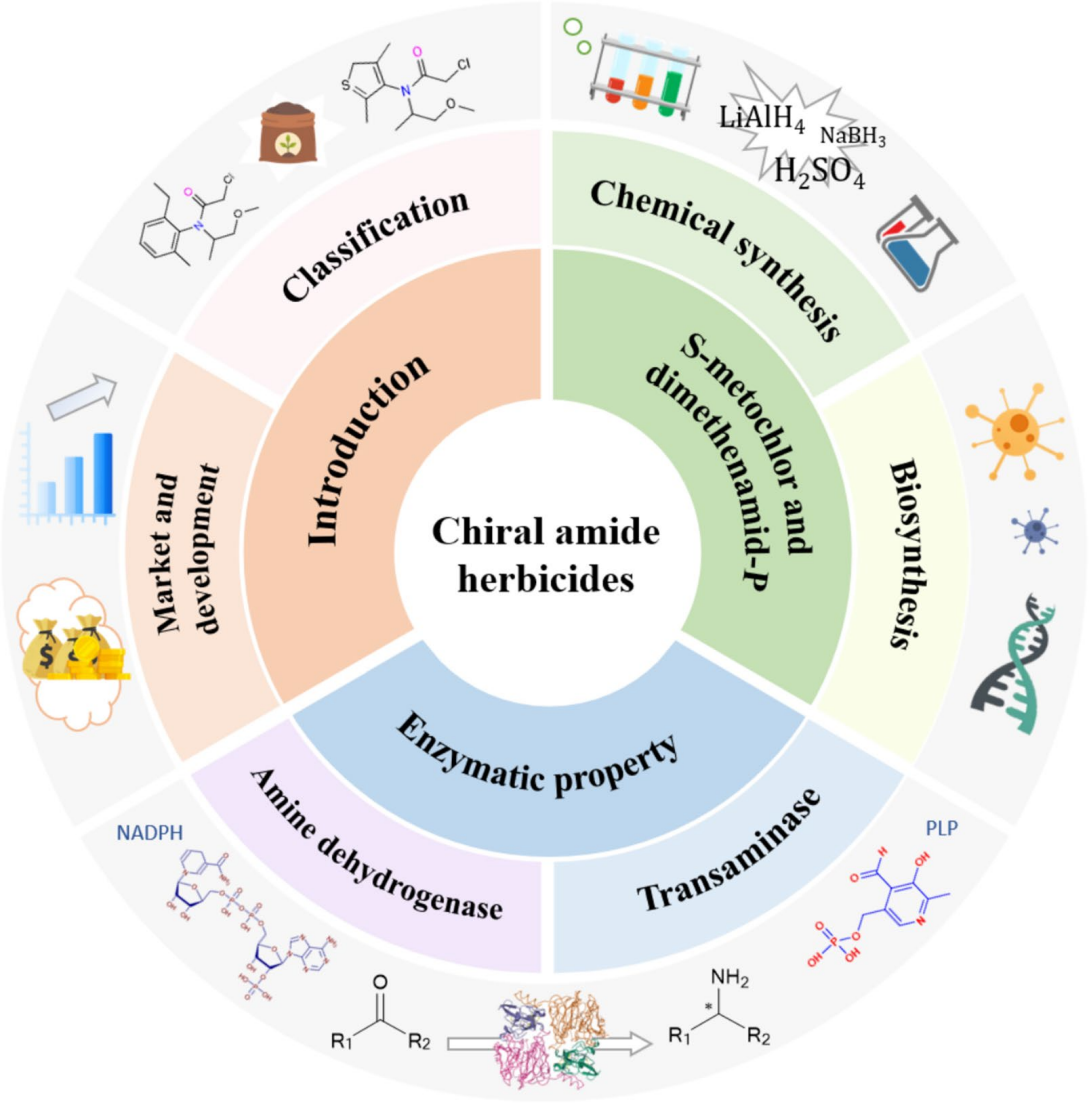
Overall framework and conceptual diagram of this review. This review is structured into three main sections: The first section offers an overview of the classification and market development of chiral amide herbicides; the second section explores the chemical synthesis and biosynthetic pathways of two major amide herbicides, S-metolachlor and dimethenamid-P; and the final section examines the enzymatic properties of two key enzymes, amine dehydrogenase and transaminase, highlighting their roles in the biosynthesis of herbicide intermediates

This review offers an overview of the classification and market trends of amide herbicides, highlighting the two major chiral amide herbicides, S-metolachlor and dimethenamid-P. It also explores their chemical and biosynthetic pathways, with a particular focus on the properties and roles of two key enzymes in biocatalysis—transaminases and amine dehydrogenases (Fig. 1).

### Overview of amide herbicides

Amide herbicides are a class of pre-emergent, broad-spectrum, selective herbicides widely used as soil treatments. These herbicides are commonly applied to crops such as soybeans, cotton, corn, and peanuts, effectively controlling annual grasses and certain broadleaf weeds like purslane and amaranth during the crop growth stage (Su 2002). They penetrate weeds through the soil, targeting roots or shoots, and subsequently inhibit essential biological processes. Specifically, they disrupt protein synthesis or fatty acid biosynthesis, thereby interfering with the formation of cellular membranes. This results in stunted growth of the weeds’ shoots and coleoptiles, eventually leading to plant death (Gao et al. 2013).

The development of amide herbicides began in 1952 when Monsanto company discovered that chloroacetamide compounds exhibited herbicidal activity. The first commercialized amide herbicide, allidochlor, was launched in 1956. In China, a country with extensive agricultural activities where staple crops like corn, rice, and soybeans constitute approximately 70% of the cultivated area, the demand for effective herbicides continues to grow. Amide herbicides have gained significant popularity due to their efficiency, broad spectrum of activity, and cost-effectiveness. Currently, they are among the most widely used herbicide categories, following amino acid herbicides (such as glyphosate, glufosinate, L-phosphinothricin) and sulfonylurea herbicides.

### Varieties and classification of amide herbicides

**Fig. 2:**
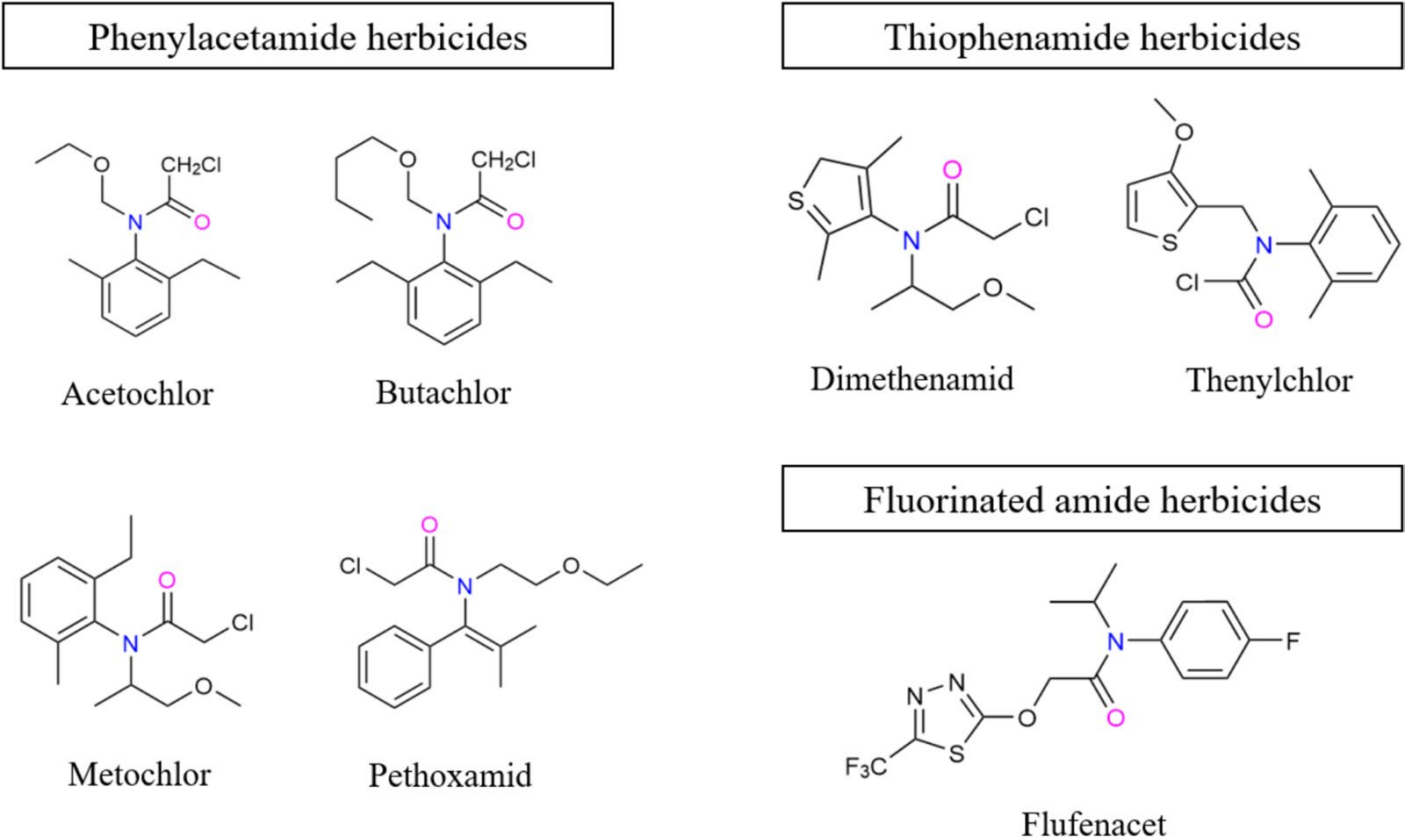
Classification of amide herbicides based on their different substituent groups. Examples include phenylacetamide herbicides, thiophenamide herbicides, and fluorinated amide herbicides

Amide herbicides can be classified based on both their chemical structure and their mechanism of action (Fig. 2). Structurally, they include chloroacetamides, sulfonamides, arylamides, alkylamides, and phenoxypropionamides. Mechanistically, they can be classified into six main types: inhibitors of protein synthesis, cell wall synthesis, cell division, fatty acid synthesis, carotenoid synthesis, and hormone-like herbicides (Zhang 2011a). Among these, inhibitors of protein and carotenoid synthesis represent the most widely used and significant subgroups. Amide herbicides have found widespread application, particularly in China, where acetochlor has been a dominant product in the domestic market since 1989. Other amide herbicides, such as metolachlor, dimethenamid, and propanil, have also been widely commercialized (Gu and Wang 2016). While these herbicides all contain an amide group (-CONH_2_), their functionality and specificity are determined by the variations in their substituents. Typically, they function as cell inhibitors, either by disrupting protein synthesis or interfering with cell division, effectively controlling weed growth (Mikula et al. 2008).

Acetochlor, developed by Monsanto in 1985, became a leading product for use in crops like corn, cotton, and soybeans. However, concerns about its potential risks to human health and its impact on surface water through persistent metabolites have led to restrictions in several countries. Another major herbicide, metolachlor, was discovered by Syngenta in 1970 and commercialized in 1975. Its more active enantiomer, S-metolachlor, was introduced in 1997, achieving significant success through formulations that combined it with other herbicides. Dimethenamid, developed by Syngenta and later patented by BASF, is another important product in this category. Its enantiomerically pure form, dimethenamid-P, has received extended regulatory approval in the European Union, reflecting its ongoing relevance. Other notable amide herbicides include thenylchlor and flufenacet. Thenylchlor, developed by Japan’s Tokuyama Corporation, is used primarily in paddy fields to control annual grasses and broadleaf weeds. Flufenacet, created by Bayer, has gained competitive advantages through its use in combination products targeting both grasses and broadleaf weeds. Despite their widespread application, these herbicides’ environmental impacts and the persistence of certain chemical residues remain critical concerns requiring further research and innovation to promote sustainable agricultural practices.

### Application market of amide herbicides

The market demand for herbicides, including amide herbicide types, has surged alongside the expansion of China’s agricultural production and cultivated land area driven by economic growth. In 2022, global herbicide sales reached $37.15 billion, accounting for nearly 50% of the total pesticide market, surpassing fungicides and insecticides, each holding approximately 25% of the market share. Between January and September 2024, China exported 1.57 million tons of herbicides, marking a 31.47% year-on-year increase. The amide herbicide industry continues to demonstrate stable growth, reflecting its integral role in agricultural practices.

Since the first amide herbicide was developed in the 1950s, numerous global companies have contributed to the research and development of different amide herbicide structures. As of now, China has registered over 2,000 amide herbicide products within their valid registration period, covering nearly 20 major varieties. However, after 2006, the introduction of new amide herbicides slowed significantly, leading to a temporary decline in sales and a deceleration of growth rates.

Despite this, the market has seen a resurgence through the launch of amide herbicide combination formulations, active isomers, and proprietary safeners, helping the sector maintain its competitiveness. From 2018 to 2023, the International Organization for Standardization (ISO) Pesticide Common Name Technical Committee approved and announced 59 new pesticides from the United States, Japan, Germany, and China, including 23 herbicides. Among these, some contain amide components such as HPPD inhibitors (e.g., iptriazopyrid and flusulfinam) and PDS inhibitors (e.g., beflubutamid-M), which are expected to enter the market in the future.

### Challenges and future development of amide herbicides

One pressing issue with amide herbicides is their toxicity to both the environment and human health. These compounds are chemically stable, highly soluble in water, and have low soil adsorption constants and long half-lives. Consequently, they can infiltrate water systems through agricultural runoff, rainfall, and atmospheric deposition during application (Liu et al. 2014). Persistent residues in soil and surface water have been identified as common pollutants, raising environmental concerns (Huang and Xiong 2009). Moreover, amide herbicides can degrade into toxic metabolites in the natural environment, which may bioaccumulate through the food chain. These byproducts can disrupt microbial and animal endocrine systems, posing significant threats to human health (Zhang 2011a). In 2008, the United States Environmental Protection Agency (USEPA) classified acetochlor and alachlor as B-2 carcinogens, while butachlor and S-metolachlor were designated as L2 and C category carcinogens, respectively (Mhadhbi and Beiras 2016). These classifications have led to usage bans in certain regions and the imposition of strict residual limits in drinking water (e.g., less than 2 μg/L for acetochlor) (Foley et al. 2008). More recently, in January 2024, the European Commission formally included S-metolachlor on its list of banned substances after multiple postponements of regulatory decisions.

Therefore, from an environmental standpoint, reducing pesticide usage while enhancing the activity of key components is critical. Chiral pesticides, which exhibit optical isomerism, offer a very promising solution. Compared to traditional pesticides, optically active pesticides offer improved efficacy while requiring lower application rates, thereby minimizing their impact on the environment and reducing risks to human health. However, the synthesis of high-activity isomers often results in the production of low-activity or inactive isomers. Consequently, the efficient synthesis of high-activity optical isomers while minimizing undesirable byproducts remains a significant research and development focus.

Among amide herbicides, dimethenamid and metolachlor are notable examples with optical isomerism. Market-preferred formulations predominantly include their enantiopure forms, such as dimethenamid-P and S-metolachlor. In recent years, researchers have been actively exploring highly selective production routes to optimize their synthesis, reflecting the industry's commitment to innovation and sustainability.

## Synthesis of dimethenamid-P

### Introduction to dimethenamid-P

Dimethenamid is a chloroacetamide herbicide with the chemical name 2-chloro-N-(2,4-dimethyl-3-thienyl)-N-(2-methoxy-1-methylethyl)acetamide. It works by inhibiting cell division, effectively preventing weed growth and achieving its herbicidal action. Originally developed by Syngenta and later commercialized by BASF, this herbicide was patented in 1982 (Zhang 2011a). As a broad-spectrum herbicide, dimethenamid is widely used on crops such as corn, soybeans, and peanuts, targeting both grass and broadleaf weeds, including species resistant to glyphosate.

**Fig. 3:**
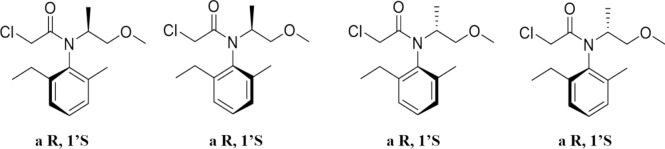
Four structural formulas of dimethenamid

The molecule of dimethenamid contains an asymmetrically substituted carbon and a chiral axis, resulting in four stereoisomers (aS,1S; aR,1S; aS,1R; and aR,1R) (Buser and Mueller 1995) (Fig. 3). Due to the low energy required for rotation around the chiral axis, the compound tends to racemize, producing two predominant diastereomers (aRS,1R and aRS,1S) used in commercial formulations. Among these, the S-enantiomers (aR,1S and aS,1S) demonstrate superior herbicidal activity and efficiency (Couderchet et al. 1997). This led to the development and commercialization of dimethenamid-P (also known as S-dimethenamid), the high-activity isomer of dimethenamid, which was patented in 1992 and introduced to the market in 2000. By 2009, dimethenamid-P achieved annual sales of $120 million in Europe and North America, with consistent growth (Wang et al. 2014). In 2019, its registration was renewed in the European Union for a 15-year period, extending its approval until September 2034.

Dimethenamid-P is recognized for its low toxicity and environmental friendliness, with no carcinogenic risks. Its effectiveness at half the dosage of the racemic mixture further underscores its efficiency. In China, its primary targets include barnyard grass and foxtail, two of the most prevalent and harmful agricultural weeds. Consequently, the domestic market demand for dimethenamid-P is substantial, highlighting the importance of developing green and efficient production methods (Liu 2005; Anderson et al. 2005).

### Chemical synthesis of dimethenamid-P

**Fig. 4:**
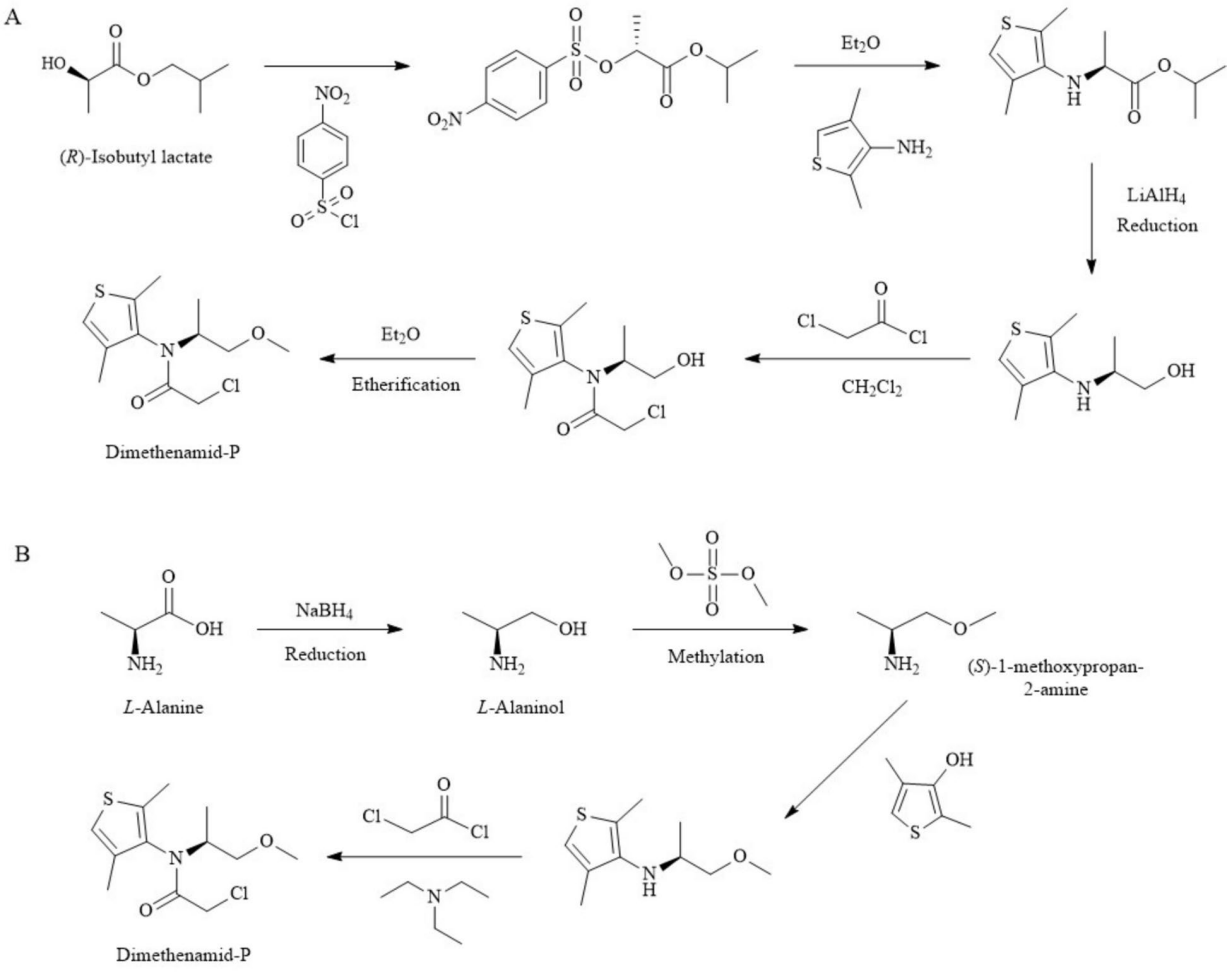
Chemical synthesis of dimethenamid-P. **A** (R)-isobutyl lactate reacts with p-nitrobenzene sulfonyl chloride and 2,4-dimethyl-3-aminothiophene to yield dimethenamid-P; **B** L-Alanine is reduced and methylated to form (S)-1-methoxypropan-2-amine, which reacts with 2,4-dimethyl-3-hydroxythiophene and chloroacetyl chloride to produce dimethenamid-P

Chemical synthesis remains the dominant approach for producing dimethenamid-P. Two synthetic routes have been detailed in existing patents, both utilizing key intermediates, 2,4-dimethyl-3-aminothiophene or 2,4-dimethyl-3-hydroxythiophene, as precursors (Fig. 4).The first route employs (R)-lactic acid isobutyl ester as the starting material, which reacts with p-nitrobenzene sulfonyl chloride to yield (R)-2-[(4-nitrophenyl) sulfonyloxy]propionic acid isobutyl ester. This intermediate undergoes reaction with 2,4-dimethyl-3-aminothiophene to produce N-(2,4-dimethyl-3-thienyl)-L-alanine isobutyl ester. Subsequent reduction with lithium aluminum hydride and reaction with chloroacetyl chloride, followed by etherification, yields dimethenamid-P. However, this route is not suitable for large-scale industrial production due to the high cost and limited availability of chiral raw materials and reducing agents, as well as the generation of significant environmental pollutants (Seckinger et al. 1995, 1987, 1983). The second route begins with L-alanine, which is reduced with sodium borohydride to form L-alaninol. Methylation with dimethyl sulfate yields (S)-1-methoxy-2-propylamine, which subsequently undergoes condensation with 2,4-dimethyl-3-hydroxythiophene and reaction with chloroacetyl chloride to produce dimethenamid-P (yield = 48%; enantiomeric excess (ee) = 85 ± 7%). Although simpler than the first route, this method still faces challenges such as the high cost of chiral starting materials, stringent reaction conditions for condensation, and difficulties in separating the desired product from its stereoisomers (Seckinger et al. 1995, 1987).

### Biosynthesis of dimethenamid-P intermediates

Agrochemicals are generally synthesized through reactions involving one or more raw materials or intermediates, making the selection of suitable intermediates critical for production (Liu et al. 2019). (S)-1-methoxy-2-propylamine is a chiral amine intermediate essential for the synthesis of the high-activity isomer dimethenamid-P. Its availability and purity significantly impact the production efficiency and application of amide herbicides like dimethenamid-P. Therefore, the efficient synthesis of high-purity (S)-1-methoxy-2-propylamine is pivotal for scaling up the production of these herbicides.

The synthesis of (S)-1-methoxy-2-propylamine can be achieved through either chemical or biological methods. However, chemical synthesis often relies on chiral substrates and is associated with low yields, environmental pollution, and high production costs. In contrast, biological synthesis presents several advantages, including reduced reliance on toxic chemical reagents, environmental sustainability, cost-effectiveness, minimized by-product formation, and fewer reaction steps. Furthermore, advancements in protein engineering enable the optimization of enzyme activity, offering tailored solutions for enhanced production efficiency.

**Fig. 5:**
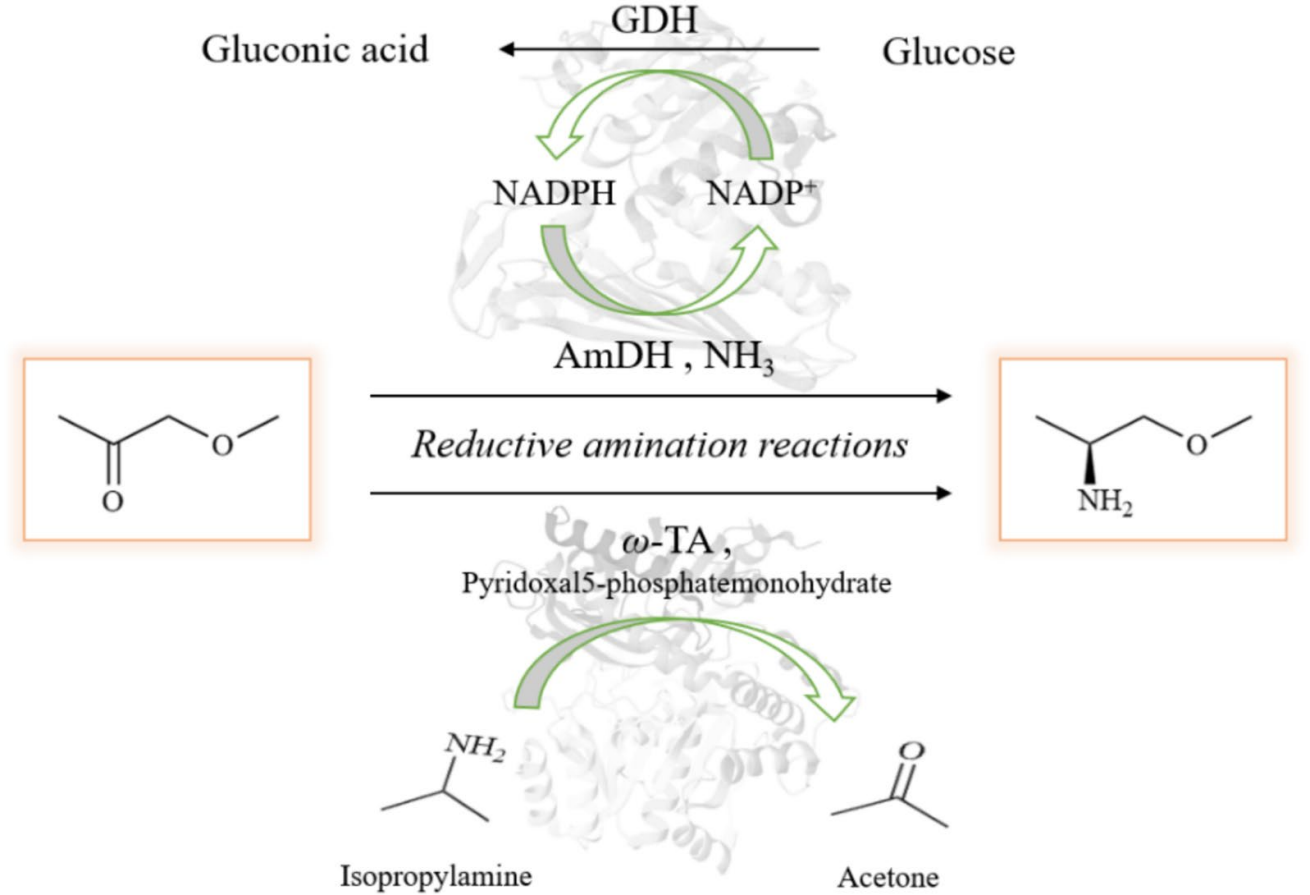
Biocatalytic synthesis of (S)-1-methoxy-2-propylamine. The two biosynthetic routes utilize amine dehydrogenase and transaminase, each within distinct biocatalytic system

Amine dehydrogenase and transaminase are currently widely used in asymmetric amination reactions, including the synthesis of (S)-1-methoxy-2-propylamine (Xue et al. 2018). These enzymes catalyze the asymmetric amination of prochiral ketone methoxyacetone as the substrate, yielding high concentrations of (S)-1-methoxy-2-propylamine. Both the substrate and product are water-soluble, facilitating the reaction process (Fig. 5). However, the equilibrium constant for transaminase-catalyzed transfer reactions is relatively low, and the conversion rate of the reversible reaction typically does not exceed 90%. On the other hand, amine dehydrogenase-catalyzed redox reactions require the supply of reductive cofactors. To improve reaction efficiency, coenzyme recycling systems are often integrated to regenerate the cofactors necessary for enzymatic activity. Recent years have seen significant advancements in the biosynthesis of (S)-1-methoxy-2-propylamine, with multiple studies reporting successful implementations of this approach (Li et al. 2023; Yang et al. 2022; Ducrot et al. 2021). These developments highlight the potential of biocatalysis as a green and efficient alternative to chemical methods for the synthesis of key chiral intermediates.

## Synthesis of S-metolachlor

Metolachlor, chemically named 2-ethyl-6-methyl-N-(1′-methyl-2′-methoxyethyl)chloride-N-acetylaniline, is one of the most widely used amide herbicides in agricultural production (Fenner et al. 2013). As a highly effective pre-emergence selective herbicide, metolachlor is suitable for weed control in over 20 crops, including corn, soybeans, peanuts, potatoes, and sugarcane. It targets annual grasses, some broadleaf weeds, and sedges (Zhao et al. 2015). The mechanism of action involves inhibiting protein synthesis in germinating seedlings, disrupting the biosynthesis of phospholipids, thereby impairing root and shoot development. Since 2011, metolachlor has surpassed acetochlor to become the most widely used amide herbicide (Sun 2013).

**Fig. 6:**
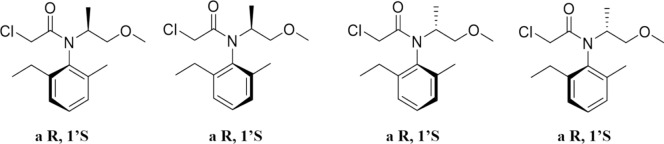
Four structural formulas of metolachlor

In 1982, Syngenta identified four stereoisomers of metolachlor and initiated activity studies on these isomers (Fig. 6). Among them, the (S)-enantiomer, marketed as S-metolachlor, exhibited superior herbicidal activity. Industrial production of S-metolachlor was realized in 1996 under the trade name Dual Gold. Currently, two main commercial forms of metolachlor are available (Zhang et al. 2015): (1) Racemic metolachlor (referred to as Dual), a mixture of R/S enantiomers. (2) S-metolachlor, which is derived from racemic metolachlor by removing the inactive or potentially mutagenic R-isomer through racemic resolution, resulting in a highly active S-isomer.

Since approximately 95% of the herbicidal activity of metolachlor originates from its S-isomer (Moser et al. 1983), S-metolachlor retains the advantageous weed control properties of metolachlor while offering superior qualities, such as higher purity, lower toxicity, and shorter residual periods. These attributes make S-metolachlor significantly more efficient and environmentally friendly compared to the racemic mixture, aligning with the principles of green pesticide development. This improvement enhances both safety and effectiveness in weed control (Chen 2016).

With the restriction or ban of acetochlor in several countries, S-metolachlor has emerged as a preferred alternative, further dominating the herbicide market. Since its introduction, its sales have steadily increased. As of January 2024, S-metolachlor has been registered in 24 technical-grade formulations and 62 preparation formulations in China. Additionally, it has secured registrations in regions including the European Union, the United States, Canada, and Australia.

### Chemical synthesis of S-metolachlor

The synthesis of S-metolachlor primarily involves three methodologies: resolution, synthesis from chiral precursors, and asymmetric catalytic hydrogenation of imines. The resolution method utilizes N-(2-methyl-6-ethylphenyl)alanine ester as a starting material, which undergoes chemical or enzymatic kinetic resolution, followed by reduction, acylation, and methylation to produce S-metolachlor (Zhang 2011b). This process is cumbersome and complex, with unstable intermediates prone to decomposition, leading to low overall yields. Due to these drawbacks, the resolution method is no longer adopted for industrial production.

In 2009, a total synthesis route using chiral reagents was developed (Zhu et al. 2009). Starting with (D)-methyl lactate or (D)-ethyl lactate, the procedure involves sequential reactions with toluene sulfonyl chloride and 2-methyl-6-ethylphenylamine, followed by reduction, chloroacylation, and methylation, ultimately yielding (S)-metolachlor. This method eliminates the need for resolution, offering a shorter reaction time and reduced costs compared to the resolution method. However, challenges such as selectivity during acylation result in suboptimal yields, limiting its applicability for large-scale industrial production.

In industrial practice, directed synthesis is often preferred, enabling the targeted production of high-purity products using chiral catalysis (Nugent et al. 1993). As the first chiral pesticide synthesized industrially using asymmetric methods, S-metolachlor has undergone extensive research. Earlier studies explored methods such as hydrogenation of enamides (Kennedy and Figueiredo 1994), nucleophilic substitution of (R)-methoxypropanol derivatives (Carlson et al. 2006), and alkylation of methoxyisopropanol (Rusek 1991). However, these approaches faced issues such as low feasibility and low conversion.

**Fig. 7:**
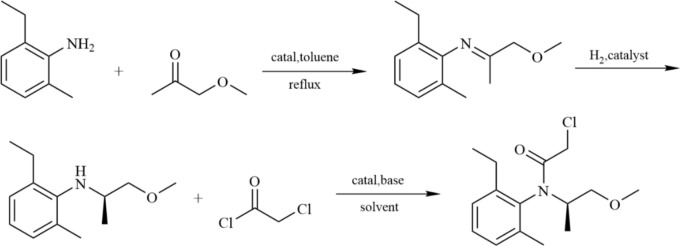
Asymmetric catalytic hydrogenation of imines for S-metolachlor synthesis

Given the cost-effectiveness and high yield of racemic intermediates, asymmetric catalytic synthesis has emerged as a viable route. Since racemic metolachlor can be prepared via reductive alkylation, asymmetric catalytic hydrogenation of imines has been considered for producing single-configuration target products. Although earlier efforts resulted in a modest ee of approximately 22% (Levi et al. 1975), significant advancements were made by Buser and colleagues (Buser et al. 2004). Utilizing a ferrocene-based diphosphine ligand catalyst, the ee value of S-metolachlor was improved to 76% and products achieved industrial-scale production. Currently, asymmetric catalytic hydrogenation of imines represents the most efficient process for producing (S)-metolachlor, as illustrated in Fig. 7 (Jing 2018).

### Biosynthesis of S-metolachlor

Chemical synthesis of S-metolachlor involves cumbersome steps, consumes a large amount of organic solvents, and produces numerous byproducts that are difficult to separate. Incomplete post-processing can lead to significant environmental pollution. Additionally, side reactions in the process can affect the optical purity of the product, making it unsuitable for long-term large-scale application. On the other hand, enzyme-catalyzed processes are highly efficient and stereoselective, operating under mild conditions, making them an ideal choice for synthesizing chiral compounds. Among enzymes, lipases are the most widely used hydrolytic enzymes, known for their high stereoselectivity towards alcohols, esters, and carboxylic acids. They are cost-effective, easy to handle, and have been extensively used in the resolution of racemic mixtures. Zheng et al. developed an enzymatic method for the resolution of N-substituted phenyl α-amino acids, using lipase-catalyzed stereoselective ester hydrolysis to prepare a chiral intermediate, 2-[(2-methyl-6-ethyl)phenylamino]propanoic acid (NEMPA), which is a key precursor to S-metolachlor. The researchers screened various lipases from different sources, including lipase from *Pseudomona*s sp. (PSL), lipase B from *Candida antarctica* (CAL-B), and lipase from *Bacillus subtilis* (BSL2) (Zheng et al. 2005). They investigated the influence of microenvironmental factors on enzyme activity and stereoselectivity, finding that enzyme activity and stereoselectivity decreased under high temperatures and slightly alkaline conditions.

**Fig. 8:**
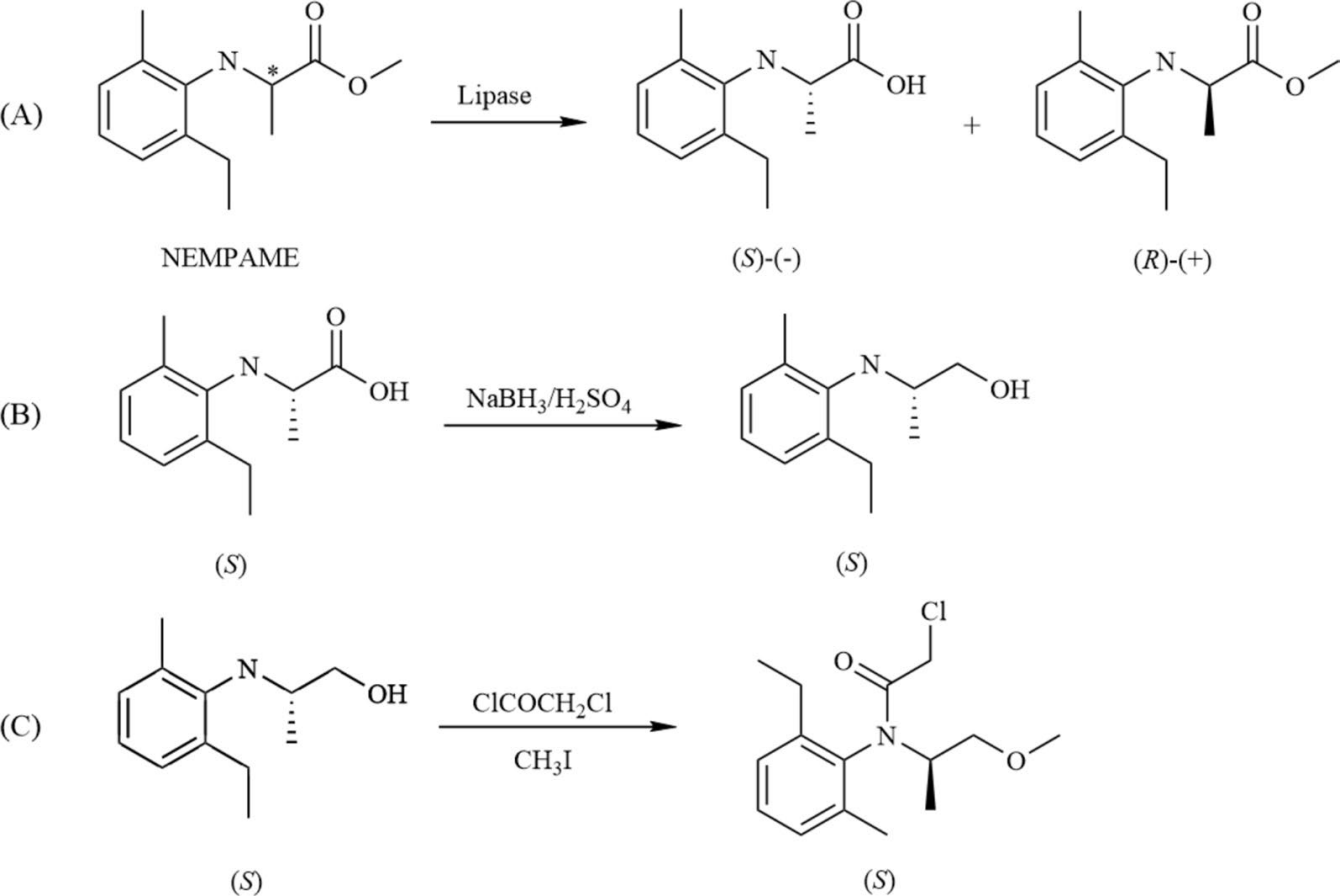
Lipase-catalyzed resolution to synthesize S-metolachlor. **A** Resolution of racemic NEMPAME using lipase through stereoselective ester hydrolysis reaction; **B** The (S)-isomer was reduced to alcohol by NaBH_3_/H_2_SO_4_ system; **C** Obtaining S-metolachlor

Furthermore, the process was found to be controlled purely by kinetics, with no substrate inhibition observed (Zheng et al. 2006). Ether solvents like diethyl ether and isopropyl ether enhanced the stereoselectivity of CAL-B and BSL2, while the surfactant Tween-80 improved stereoselectivity for both CAL-B and BSL2, though it only benefited CAL-B in terms of catalytic activity. Additionally, PSL exhibited better stereoselectivity than CAL-B for the chiral intermediate 2-[(2-methyl-6-ethyl)phenylamino]propanoic acid. Following this, S-metolachlor was successfully synthesized via a three-step synthetic process using PSL, achieving the desired product with high stereoisomeric purity (Fig. 8). Although lipase-based biosynthesis has been successful, the maximum product yield from kinetic resolution is only 50%, which significantly limits its further industrial application. Thus, after a simple extraction procedure is used to separate the acid product from the remaining ester, the remaining ester is racemized, providing the basis for the continuous resolution process (Zheng et al. 2006). Furthermore, the transamination method can achieve the theoretical maximum yield of 100%, making it an ideal synthesis process. This process is discussed in detail in the following sections.

## Key enzyme creation and engineering

Over the past decade, significant progress has been made in the field of enzymatic synthesis of chiral amines, driven by their importance in various industries, such as the synthesis of sitagliptin using transaminases. Additionally, new enzymes have been developed for catalyzing enantioselective amine reactions. This section discusses the catalytic processes of amine dehydrogenases (AmDHs) and transaminases, focusing on their role in the synthesis of the common intermediate (S)-1-methoxy-2-propylamine, used for the production of S-metolachlor and dimethenamid-P.

### Amine dehydrogenase

**Fig. 9:**
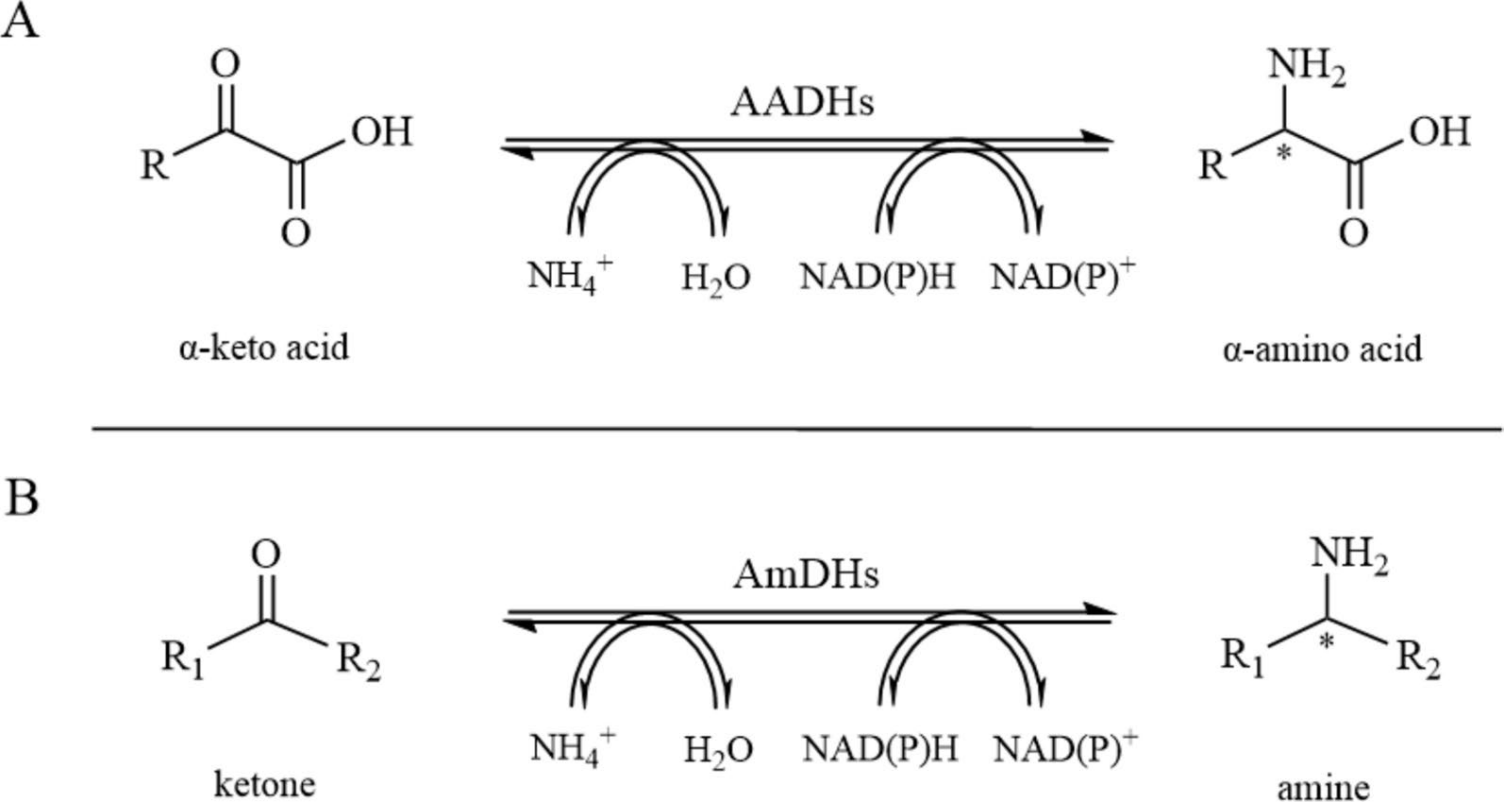
Reductive amination of α-keto acid and ketone by oxidoreductase. **A** Reaction catalyzed by amino acid dehydrogenase; **B** Reaction catalyzed by amine dehydrogenase

Amine dehydrogenases (AmDHs) use ammonia as their amino donor, catalyzing asymmetric amination reactions with aldehyde and ketone substrates. The sole byproduct is water. Due to the low cost of ammonia as an amino donor, the reactions exhibit high atomic efficiency and enantioselectivity, with simple product purification, making them highly promising for chiral amine synthesis applications (Jeon et al. 2017) (Fig. 9).

Since AmDHs are relatively rare in nature, they can be obtained through two main approaches: engineering existing amino acid dehydrogenases (AADH) to function as AmDH, or discovering new natural amine dehydrogenases from a variety of microorganisms (Lee et al. 2021). In 2012, Abrahamson et al. (Abrahamson et al. 2012) reported the first engineered AmDH, followed by extensive research leading to the development of engineered AmDHs based on other amino acid dehydrogenases, such as leucine dehydrogenase (Chen et al. 2015), phenylalanine dehydrogenase (Ye et al. 2015), and lysine dehydrogenase (Tseliou et al. 2019). However, the substrate scope limitation has hindered the broader application of AADHs (Schrittwieser et al. 2015). As a result, researchers began to search for more versatile AmDHs, which can also catalyze the reductive amination of ketones with ammonia, have broader substrate specificity compared to AADHs, making them more suitable for various applications. In recent years, natural AmDHs have been discovered in various bacteria and eukaryotic organisms, allowing for enzyme engineering of wild-type AmDHs to obtain enzymes with superior performance (Table 1). In 1999, Itoh et al.reported a novel AmDH from *Streptococcus viridans* IFO 12827, which is NADH-dependent and can catalyze the reductive amination of a wide range of substrates, producing various amino alcohols and amine derivatives from aldehydes, ketones, keto acids, and ketols (Itoh et al. 2000). This enzyme exhibited maximum reductive amination activity at pH 6.5–7.0 and 25 °C. However, due to low stereoselectivity and poor reproducibility, the natural AmDH library was not expanded until the discovery of the first usable (S)-specific enzyme.

**Table 1:** Recently discovered amine dehydrogenases from different sources for the biotransformation of ketones into chiral amines

Substrate	Product	Organism	Enzyme	Conversion(%);*ee*(%)	References
		*Mycolicibacterium smegmatis*	*Msme*AmDH	44.7; > 93(S)	(Ducrot et al. 2021)
		*Microbacterium sp.*	*Micro*AmDH	35.3; > 81.9(S)	(Ducrot et al. 2021)
		*Geobacillus kaustophilus*	*Gk*AmDH	77; > 99(S)	(Liu et al. 2020)
		*Geobacillus kaustophilus*	*Gk*AmDH	99; > 99(S)	(Liu et al. 2020)
		*Mycolicibacterium smegmatis*	*Msme*AmDH	87.4; > 90(S)	(Ducrot et al. 2021)
		*Microbacterium sp.*	*Micro*AmDH	97.1; > 91(S)	(Ducrot et al. 2021)
		*Lysinibacillus sphaericus* CBAM5	*Ls*AmDH	63; > 99(S)	(Wang et al. 2020)
		*Streptococcus viridans*	*Sv*AmDH	–; > 99(R)	(Itoh et al. 2000)
		*Cystobacter fuscus*	*Cfus*AmDH	78.8; > 99(S)	(Ducrot et al. 2021)
		*Escherichia coli metagenome*	MATOU AmDH2	75.7; > 96(S)	(Ducrot et al. 2021)
		*Geobacillus stearothermophilus*	*Gs*AmDH	36; > 99(R)	(Wang et al. 2020)
		*Geobacillus stearothermophilus*	*Gs*AmDH	99; > 99(S)	(Wang et al. 2020)
		*Lysinibacillus sphaericus* CBAM5	*Ls*AmDH	95; > 99(S)	(Wang et al. 2020)
		*Bacillus stearothermophilus*	*Bs*AmDH	94; > 99(S)	(Wang et al. 2020)
		*Sporosarcina psychrophile*	*Sp*AmDH	79; > 99(S)	(Wang et al. 2020)
		*Petrotoga mobilis* DSM 10674	*Pm*AmDH	90; > 99.5(S)	(Mayol et al. 2016)
		*Escherichia coli metagenome*	MATOUAmDH2	41; N.D	(Bennett et al. 2022)

In 2016, Vergne-Vaxelaire’s research group discovered a heat-stable AmDH, AmDH4, from *Petrotoga mobilis* DSM 10674 (Mayol et al. 2016). This enzyme efficiently catalyzed the reductive amination of ketones without carboxyl groups at the α or β positions, converting 4-keto-5-acid to (4S)-4-amino-5-acid with high stereoselectivity (ee ≥ 99.5%) and an 88% yield. This was the first report of a wild-type NAD(P)H-dependent AmDH with excellent stereoselectivity. With the rise of genomic mining and metagenomics technologies, more natural AmDH genes have been discovered, further expanding the AmDH enzyme family (Caparco et al. 2020; Mayol et al. 2019).

Ducrot et al. (2021) investigated the potential of natural AmDHs, including *Cfus*AmDH, *Msme*AmDH, *Micro*AmDH, and MATOUAmDH2, for biocatalytic synthesis of short fatty amines and hydroxylamines, focusing on the conversion from methoxyacetone to (S)-1-methoxy-2-propylamine. At a 10 mM substrate concentration, the conversion rates for methoxyacetone were 63.9%, 56.5%, 78.4%, and 30.0% for *Cfus*AmDH, *Msme*AmDH, *Micro*AmDH, and MATOUAmDH2, respectively. When the substrate concentration was increased to 50 mM, significant improvements in conversion rates were observed, with *Micro*AmDH reaching a conversion rate of 98.1%. Additionally, semi-preparative scale-up experiments were successfully conducted at a 150 mM substrate concentration, demonstrating the feasibility of synthesizing (S)-but-2-amine and (S)-1-methoxy-2-propylamine. Among these, *Msme*AmDH was identified as the most promising enzyme for catalyzing the conversion of methoxyacetone to (S)-1-methoxy-2-propylamine, achieving a conversion rate of 88.3% and an enantiomeric excess (ee) of 98.6%. Although product separation was not performed due to the high solubility and volatility of the product, the industrial synthesis route for (S)-1-methoxy-2-propylamine proved the feasibility of this process step.

### Coenzyme regeneration system for amine dehydrogenases

Coenzymes play a crucial role as hydrogen proton donors in enzyme-catalyzed reactions. Redox enzymes, which are widely used in industrial applications, rely on cofactors such as nicotinamide-based cofactors (NAD⁺/NADH or their phosphorylated forms NADP⁺/NADPH), flavin cofactors (FAD/FMN), coenzyme Q, and heme. Amine dehydrogenases require continuous supply of NAD(P)H during the reaction. However, the high cost of natural nicotinamide-based cofactors makes their large-scale industrial use impractical. To enhance the economic viability of amine dehydrogenase applications, it is essential to develop coenzyme regeneration systems that can efficiently recycle oxidized NAD(P)⁺ to its reduced form NAD(P)H.

**Fig. 10:**
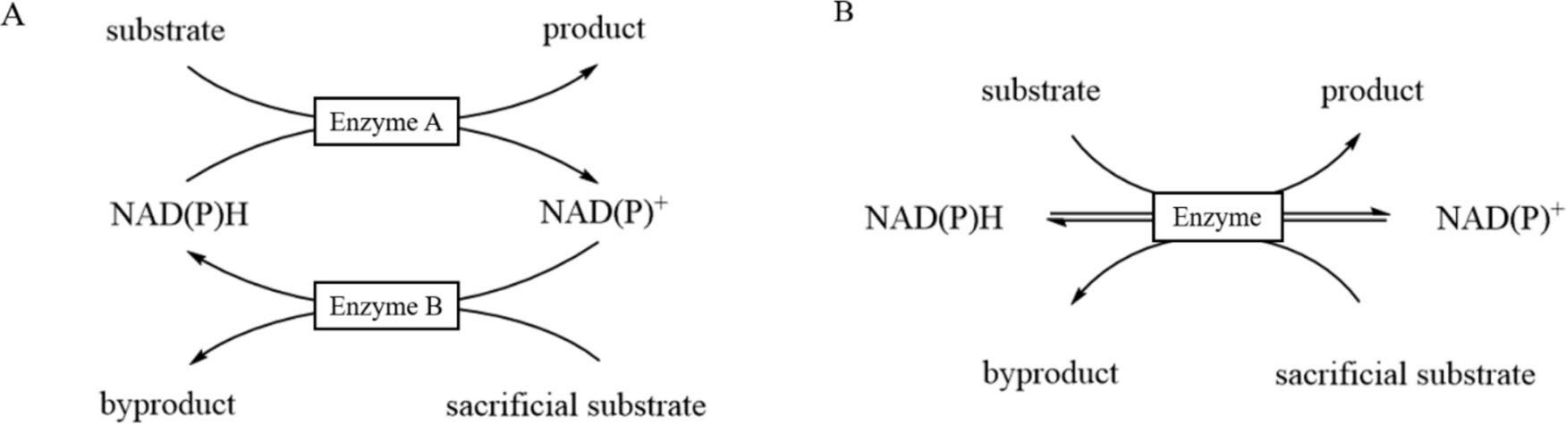
Commonly enzymatic-based cofactor regeneration. **A** Enzyme-coupled method; **B** Substrate-coupled method

Several coenzyme regeneration methods have been developed, including chemical, electrochemical, photocatalytic, and enzymatic approaches (Yuan et al. 2019; Brown et al. 2016). Among these, enzymatic regeneration is commonly used for coenzyme recycling in amine dehydrogenase catalysis. Enzymatic regeneration can be classified into substrate-coupled and enzyme-coupled methods. In the substrate-coupled method, a secondary substrate is added to the enzyme system, enabling the enzyme to catalyze both oxidation and reduction reactions, thus facilitating the consumption and regeneration of the cofactor. In the enzyme-coupled method, both oxidation and reduction enzymes are introduced into the reaction system, allowing the auxiliary enzyme to generate the required cofactor in parallel with the main reaction, ensuring continuous supply of the cofactor for the main enzyme reaction (Fig. 10).

Amine dehydrogenases (AmDHs) often rely on the enzyme-coupling method for catalysis, where the enzyme catalyzes the reduction of ketones to amines while simultaneously oxidizing NAD(P)H to NAD(P)⁺. This process is coupled with another enzyme that catalyzes the dehydrogenation of the corresponding substrate, converting NAD(P)⁺ back into NAD(P)H. Both reactions occur in tandem, enabling the main reaction to proceed without requiring external cofactor supply. This coupled system facilitates the recycling of NAD(P)H and NAD(P)⁺, allowing for a one-pot reaction system where cofactors are continually regenerated, enhancing the overall efficiency of the reaction (Wang et al. 2020).

**Fig. 11:**
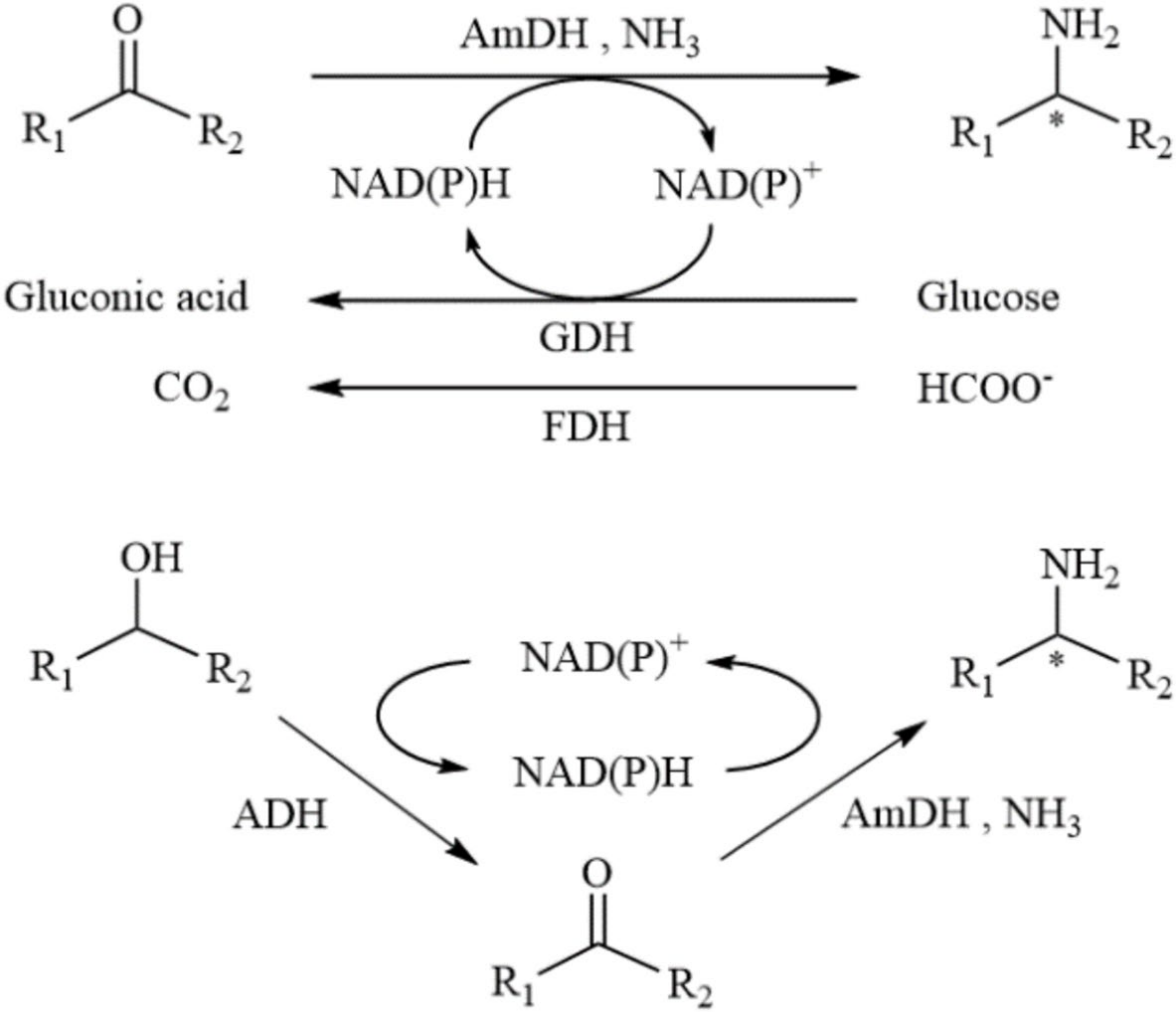
The reaction system of amine dehydrogenase coupled with other enzymes

Constructing an effective coenzyme regeneration system involves various factors such as enzyme stability, the rate matching of reactions in the system, and efficient product separation and purification (Hummel and Riebel 2003). To optimize this process, enzymes that consume NAD(P)⁺, like glucose dehydrogenase (GDH), formate dehydrogenase (FDH), and alcohol dehydrogenase (ADH), are often used in cascade reactions with AmDHs (Cheng et al. 2024; Mutti et al. 2015) (Fig. 11). GDH is particularly useful for regenerating NADH and NADPH, as it uses glucose, an inexpensive and easily available substrate, to produce gluconic acid, a non-toxic oxidation product. This system is highly efficient and environmentally friendly, with no adverse effects on enzyme performance or product recovery, making it a preferred method for coenzyme regeneration in bioprocesses.

Formate dehydrogenase (FDH), which is highly specific for formate as a substrate, is another commonly used enzyme for NADH regeneration. The resulting CO₂ by-product does not interfere with most reaction systems, and it can be easily removed in a closed reaction setup, making FDH particularly suitable for reactions that require large amounts of NADH (Marchini et al. 2022). Similarly, alcohol dehydrogenase (ADH) can convert alcohols like ethanol or isopropanol into aldehydes, generating NADH or NADPH in the process. This versatile enzyme is also commonly used for coenzyme regeneration in various biocatalytic reactions. Mutti et al. developed a dual-enzyme system that coupled alcohol dehydrogenase and amine dehydrogenase, where the ketone product from the alcohol dehydrogenase reaction serves as the substrate for the amine dehydrogenase (Mutti et al. 2015). In this setup, only an external amine donor is required, with water as the sole by-product. This self-sufficient system enables efficient amination reactions while maintaining a continuous supply of cofactors.

### Transaminase

In recent years, biocatalytic transamination reactions have attracted increasing market attention, as chiral amine products generated through biocatalysis are key components in the pharmaceutical industry. Transamination reactions can occur through either the kinetic resolution of racemic amines or the asymmetric synthesis of prochiral ketones, and they do not require the regeneration of expensive cofactors. Among these, asymmetric synthesis has become a more common method due to its higher theoretical yields (Rehn et al. 2016). A notable example is the work reported by Savile et al. in 2010, where ω-transaminase was used in the biocatalytic production of sitagliptin, replacing the previous rhodium-catalyzed asymmetric olefin amine hydrogenation method (Savile et al. 2010). This demonstrated that ω-transaminases could serve as powerful catalysts for the asymmetric synthesis of α-chiral primary amines, marking a significant advancement in the production of the anti-diabetic drug sitagliptin.

In 1990, Celgene developed a proprietary biocatalytic transamination technology capable of converting prochiral ketones into high optical purity enantiomers of chiral amines. Following this, amino donors such as isopropylamine (IPA) began to be utilized in the production of chiral amines. IPA, a non-chiral reagent, is thermodynamically advantageous for most reactions. It is also cost-effective, readily available, and the conversion product, acetone, is easily recoverable and commercially valuable, making it an efficient and practical choice for large-scale amine synthesis (Matcham et al. 1999).

**Fig. 12:**
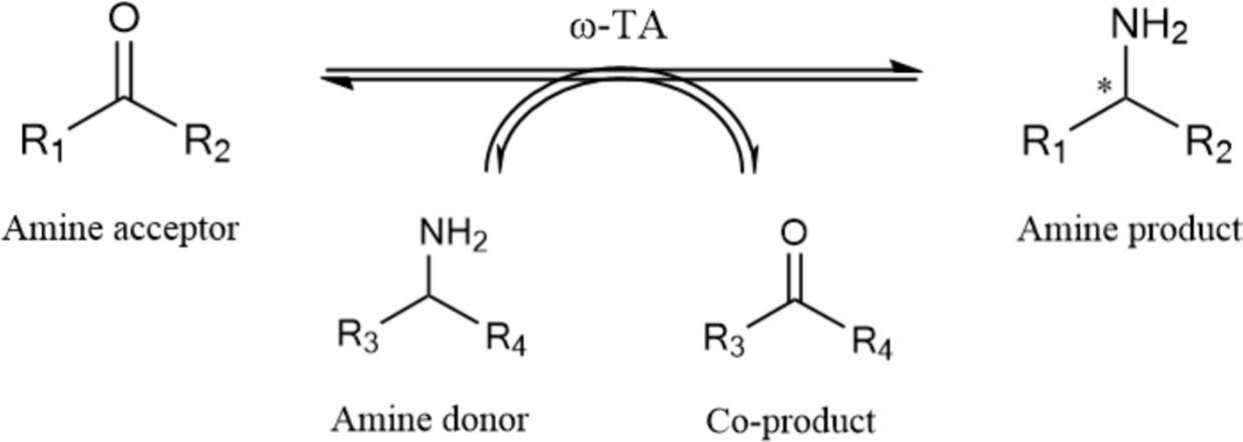
Synthesis of (S) -1-methoxy-2-propylamine catalyzed by transaminase

Transaminases are enzymes that catalyze the reversible transfer of an amino group between a variety of amino compounds, such as amino acids, aliphatic amines, and aromatic amines, and carbonyl compounds, including aldehydes, ketones, and keto acids (Li et al. 2023) (Table 2). These enzymes are highly valued for their broad substrate range, high stereoselectivity, and mild catalytic conditions, making them ideal for the green biosynthesis of chiral amines. For instance, transaminases can catalyze the conversion of 1-methoxy-2-propanone to (*S*)-1-methoxy-2-propylamine with high optical purity under simple, mild conditions. In 2001, a patent disclosed a method in which transaminase catalyzed the conversion of methoxyacetone to (*S*)-1-methoxy-2-propylamine using isopropylamine as the amino donor, yielding acetone as a by-product, which could be easily recovered (Wu et al. 2001) (Fig. 12). However, this patent did not disclose the source or gene sequence of the transaminase used, limiting the potential for subsequent research and application.

**Table 2:** Enzymatic properties of recently discovered transaminases from different sources

Substrate	Product	Organism	Enzyme	K_m_ (mM)	k_cat_/K_m_ (mM^−1^ s^−1^)	References
		*Arthrobacter sp.*	*As*TA	0.24 ± 0.03	15.49 ± 0.29	(Liu et al. 2024)
		*Bacillus soil 768D1*	*Bs*TA	–	–	(Yang et al. 2022)
		*Vibrio fluvialis*	*Vf*TA	8.59 + 0.18	0.013	(Midelfort et al. 2013)
		*Burkholderia vietnamiensis G4*	*Bv*TA	12.9 ± 0.9	1.6	(Wang et al. 2021)
		*Aspergillus fumigatus*	*Af*TA	0.61 ± 0.04	18.21	(Xiang et al. 2022)
		*Vibrio fluvialis*	*Vf*Ta	0.155	4.3*10^4^	(Dourado et al. 2016)
		*Mycobacterium vanbaalenii*	*Mv*Ta	0.19 ± 0.012	34.47	(Cheng et al. 2020)
		*Mycolicibacterium wolinskyi*	*Mw*Ta	8.89	5.45	(Zhu et al. 2023)

For example, Yang et al. discovered a wild-type transaminase from *Bacillus* Soil768D1, which showed high amination activity when treated with 0.15–10 M isopropylamine or its salts, yielding a product with an enantiomeric excess (ee) of over 99% (Yang et al. 2022). They further engineered the enzyme via site-directed mutagenesis, resulting in improved enzyme activity and conversion rates. The reaction required the addition of co-factors such as pyridoxal phosphate, pyridoxamine phosphate, or pyridoxal 5′-phosphate, at concentrations ranging from 0.1 to 100 mM, to assist the transaminase in catalysis. This method offers a simple process with mild reaction conditions, making it suitable for the green, cost-effective production of (S)-1-methoxy-2-propylamine.

Transaminase-catalyzed reactions, due to their reversibility, can influence the yield of the desired product. Therefore, it is essential to adopt appropriate strategies to remove carbonyl by-products to prevent the reverse reaction from competing and impacting the conversion rate (Guo and Berglund 2017; Gundersen et al. 2015). Common amino donors, such as alanine and isopropylamine, generate by-products like pyruvate and acetone, which can interfere with the reaction. To control the reaction direction, high concentrations of isopropylamine can be added to suppress the reverse reaction. This requires enzymes to tolerate high substrate concentrations, a critical consideration for enzyme engineering. Additionally, developing strategies to remove or recover by-products like acetone remains an important area for further research.

## Summary and outlook

This review explores the classification and market development of amide herbicides, with a focus on the two prominent examples, S-metolachlor and dimethenamid-P. It analyzes and summarizes their chemical and biological synthesis routes, emphasizing the role of biocatalysts—transaminases and amine dehydrogenases—in the production of their common intermediate, (S)-1-methoxy-2-propylamine. Additionally, it provides insights into the processes and strategies that could be leveraged for the biocatalytic synthesis of similar agricultural chemicals.

Over the years, amide herbicides have maintained a significant share in the global herbicide and pesticide markets. They have been widely used as one of the most popular herbicides, and innovation in their development has remained a focal point of research. However, since the early twenty-first century, no new amide herbicides have been introduced to the market. With the development of more efficient and safer herbicides, the growth rate of amide herbicides has slowed. Metochlor and Dimethenamid, as key types of amide herbicides, both exist in highly effective optical isomer forms, traditionally synthesized through chemical methods. In contrast, biocatalytic methods, due to their advantages of high activity, high yield, and simple processes, are gradually overcoming the limitations of chemical synthesis and gaining traction in the industrial production of pesticides, especially in the production of chiral pesticides. As mentioned, (S)-1-methoxy-2-propylamine, as an intermediate, plays an essential role in the production of S-metochlor and Dimethenamid-P, holding significant importance for pesticide manufacturing.

Currently, research into the biocatalytic synthesis of (S)-1-methoxy-2-propylamine is still limited, presenting a promising area for further development. Moreover, the transaminases and amine dehydrogenases involved in its synthesis are crucial oxidoreductases in chiral amine production. Notably, over the past decade, significant progress has been made in the enzyme-catalyzed synthesis of chiral amines, demonstrating the importance of enzyme design and modification for future research in chiral amine-based pesticides. The development of biocatalysts for chiral amines will play a key role in advancing the green synthesis of agricultural chemicals.

## Data Availability

Not applicable.

## References

[CR1] Abrahamson MJ, Vázquez-Figueroa E, Woodall NB, Moore JC, Bommarius AS (2012) Development of an amine dehydrogenase for synthesis of chiral amines. Angew Chem Int Ed 51(16):3969–3972. 10.1002/anie.201107813 10.1002/anie.201107813 22396126

[CR2] Anderson KA, Basile JL, Johnson ER (2005) Analytical method for dimethenamid-P in selected raw agricultural commodities by gas chromatography with electron capture detection. J AOAC Int 88(5):1428–1432. 10.1093/jaoac/88.5.1428 16385993

[CR3] Bennett M, Ducrot L, Vergne-Vaxelaire C, Grogan G (2022) Structure and mutation of the native amine dehydrogenase MATOUAmDH2. ChemBioChem 23(10):e202200136. 10.1002/cbic.202200136 35349204 10.1002/cbic.202200136 PMC9325545

[CR4] Brown KA, Wilker MB, Boehm M, Hamby H, Dukovic G, King PW (2016) Photocatalytic regeneration of nicotinamide cofactors by quantum dot-enzyme biohybrid complexes. ACS Catal 6(4):2201–2204. 10.1021/acscatal.5b02850

[CR5] Buser HR, Mueller MD (1995) Environmental behavior of acetamide pesticide stereoisomers 1 Stereo- and enantioselective determination using chiral high-resolution gas chromatography and chiral HPLC. Environ Sci Technol 29(8):2023–2030. 10.1021/es00008a022 22191351 10.1021/es00008a022

[CR6] Buser H-P, Hübner F, Müller M, Spindler F, Wuhrmann S (2004) Process for the hydrogenation of imines. US Patent 6822118B1.

[CR7] Caparco AA, Pelletier E, Petit JL, Jouenne A, Bommarius BR, De Berardinis V, Zaparucha A, Champion JA, Bommarius AS, Vergne-Vaxelaire C (2020) Metagenomic mining for amine dehydrogenase discovery. Adv Synth Catal 362(12):2427–2436. 10.1002/adsc.202000094

[CR8] Carlson DL, Than KD, Roberts AL (2006) Acid- and base-catalyzed hydrolysis of chloroacetamide herbicides. J Agric Food Chem 54(13):4740–4750. 10.1021/jf0530704 16787023 10.1021/jf0530704

[CR9] Chen YL (2016) S-Metolachlor, a chloroacetamide herbicide. Mod Agrochem 15(3):40–43. 10.3969/j.issn.1671-5284.2016.03.013

[CR10] Chen FF, Liu YY, Zheng GW, Xu JH (2015) Asymmetric amination of secondary alcohols by using a redox-neutral two-enzyme cascade. ChemCatChem 7(23):3838–3841. 10.1002/cctc.201500785

[CR11] Cheng F, Chen XL, Xiang C, Liu ZQ, Wang YJ, Zheng YG (2020) Fluorescence-based high-throughput screening system for R-ω-transaminase engineering and its substrate scope extension. Appl Microbiol Biotechnol 104(7):2999–3009. 10.1007/s00253-020-10444-y 32064550 10.1007/s00253-020-10444-y

[CR12] Cheng F, Wang CJ, Gong XX, Sun KX, Liang XH, Xue YP, Zheng YG (2024) Assembly and engineering of BioBricks to develop an efficient NADH regeneration system. Appl Environ Microbiol 90(4):e01041-e1124. 10.1128/aem.01041-24 10.1128/aem.01041-24 PMC11784351 39660873

[CR13] Couderchet M, Bocion PF, Chollet R, Seckinger K, Böger P (1997) Biological activity of two stereoisomers of the N-thienyl chloroacetamide herbicide dimethenamid. Pestic Sci 50(3):221–227. 10.1002/(SICI)1096-9063(199707)50:3%3c221::AID-PS574%3e3.0.CO;2-T

[CR14] Cui X, Zhong Q, Zhang XZ, Li HX, Wang XR, Luo FJ, Cheng YP, Chen ZM (2019) Progress on analysis of chiral pesticide enantiomers residues in agricultural products based on chromatographic method. J Instrum Anal 38(2):249–262. 10.3969/j.issn.1004-4957.2019.02.020

[CR15] Dourado DFAR, Pohle S, Carvalho ATP, Dheeman DS, Caswell JM, Skvortsov T, Miskelly I, Brown RT, Quinn DJ, Allen CCR, Kulakov L, Huang M, Moody TS (2016) Rational design of a (S)-selective-transaminase for asymmetric synthesis of (1S)-1-(1,1′-biphenyl-2-yl)ethanamine. ACS Catal 6(11):7749–7759. 10.1021/acscatal.6b02380

[CR16] Drăghici C, Chirila E, Sica M (2013) Enantioselectivity of chiral pesticides in the environment. Environ Secur Assess Manag Obsol Pestic S E Eur 965:91–102. 10.1007/978-94-007-6461-3_7

[CR17] Ducrot L, Bennett M, Caparco AA, Champion JA, Bommarius AS, Zaparucha A, Grogan G, Vergne-Vaxelaire C (2021) Biocatalytic reductive amination by native amine dehydrogenases to access short chiral alkyl amines and amino alcohols. Front Catal 1:781284. 10.3389/fctls.2021.781284

[CR18] Fenner K, Canonica S, Wackett LP, Elsner M (2013) Evaluating pesticide degradation in the environment: blind spots and emerging opportunities. Science 341(6147):752–758. 10.1126/science.1236281 23950532 10.1126/science.1236281

[CR19] Foley ME, Sigler V, Gruden CL (2008) A multiphasic characterization of the impact of the herbicide acetochlor on freshwater bacterial communities. ISME J 2(1):56–66. 10.1038/ismej.2007.99 18180747 10.1038/ismej.2007.99

[CR20] Gao JD, Tian XS, Feng L, Yang CH, Yue MF, Cui Y, Zhang TJ (2013) The action mechanism and advancement about amide herbicide safeners. Guangdong Agric Sci 40(22):101–105. 10.16768/j.issn.1004-874x.2013.22.023

[CR21] Gu LL, Wang XX (2016) The global market, development, trend of herbicide (I). Mod Agrochem 15(2):8–12. 10.3969/j.issn.1671-5284.2016.02.002

[CR22] Gundersen MT, Abu R, Schürmann M, Woodley JM (2015) Amine donor and acceptor influence on the thermodynamics of ω-transaminase reactions. Tetrahedron Asymmetry 26(10–11):567–570. 10.1016/j.tetasy.2015.04.006

[CR23] Guo F, Berglund P (2017) Transaminase biocatalysis: optimization and application. Green Chem 19(2):333–360. 10.1039/c6gc02328b

[CR24] Huang H, Xiong ZT (2009) Toxic effects of cadmium, acetochlor and bensulfuron-methyl on nitrogen metabolism and plant growth in rice seedlings. Pestic Biochem Physiol 94(2):64–67. 10.1016/j.pestbp.2009.04.003

[CR25] Hummel W, Riebel B (2003) Isolation and biochemical characterization of a new NADH oxidase from Lactobacillus brevis. Biotechnol Lett 25(1):51–54. 10.1023/A:1021730131633 12882306 10.1023/a:1021730131633

[CR26] Itoh N, Yachi C, Kudome T (2000) Determining a novel NAD+-dependent amine dehydrogenase with a broad substrate range from *Streptomyces virginiae* IFO 12827: purification and characterization. J Mol Catal B Enzym 10(1):281–290. 10.1016/S1381-1177(00)00111-9

[CR27] Jeon H, Yoon S, Ahsan MM, Sung S, Kim GH, Sundaramoorthy U, Rhee SK, Yun H (2017) The kinetic resolution of racemic amines using a whole-cell biocatalyst co-expressing amine dehydrogenase and NADH oxidase. Catalysts 7(9):251. 10.3390/catal7090251

[CR28] Jing WH (2018) Study on the synthesis of herbicide product S-metolachlor. Dissertation, Zhejiang University of Technology.

[CR29] Kennedy JF, Figueiredo ZMB (1994) Chirality in industry — The commercial manufacture and applications of optically active compounds. Carbohydr Polym 23(1):76. 10.1016/0144-8617(94)90095-7

[CR30] Lee S, Jeon H, Giri P, Lee UJ, Jung H, Lim S, Sarak S, Khobragade TP, Kim BG, Yun H (2021) The reductive amination of carbonyl compounds using native amine dehydrogenase from *Laribacter hongkongensis*. Biotechnol Bioprocess Eng 26(3):384–391. 10.1007/s12257-021-0113-2

[CR31] Levi A, Modena G, Scorrano G (1975) Asymmetric reduction of carbon–nitrogen, carbon–oxygen, and carbon–carbon double bonds by homogeneous catalytic hydrogenation. Chem Inform 6(13):chin.197513169. 10.1002/chin.197513169

[CR32] Li ZX, Liu Y, Luo Q, Lü XF (2023) The advance of ω-transaminase in chiral amine biosynthesis in China from the perspective of patents. Chin J Biotechnol 39(8):3169–3187. 10.13345/j.cjb.220908 10.13345/j.cjb.220908 37622354

[CR33] Liu HJ (2005) Biochemical behavior and enantioselectivity of acetanilide herbicides. Dissertation, Zhejiang University.

[CR34] Liu Y, Hu Y, Jiang L, Pan B, Qin HC, Lin Y (2014) The toxicity effects of five amide herbicides on embryo development of zebrafish. Agrochem 53(11):806–808. 10.16820/j.cnki.1006-0413.2014.11.008

[CR35] Liu CL, Guan AY, Li M, Yang JC (2019) The intermediate derivatization method and novel agrochemical discovery. Agrochem 58(3):157–164. 10.16820/j.cnki.1006-0413.2019.03.001

[CR36] Liu L, Wang DH, Chen FF, Zhang ZJ, Chen Q, Xu JH, Wang ZL, Zheng GW (2020) Development of an engineered thermostable amine dehydrogenase for the synthesis of structurally diverse chiral amines. Catal Sci Technol 10(8):2353–2358. 10.1039/D0CY00071J

[CR37] Liu H, Gao Q, Zhang K, Xu M, Wang H, Wei D (2024) Combining binding pocket mutagenesis and substrate tunnel engineering to improve an (R)-selective transaminase for the efficient synthesis of (R)-3-aminobutanol. Biochem Biophys Res Commun 731:150383. 10.1016/j.bbrc.2024.150383 39024977 10.1016/j.bbrc.2024.150383

[CR38] Marchini V, Benítez-Mateos AI, Hutter SL, Paradisi F (2022) Fusion of formate dehydrogenase and alanine dehydrogenase as an amino donor regenerating system coupled to transaminases. ChemBioChem 23(21):e202200428. 10.1002/cbic.202200428 36066500 10.1002/cbic.202200428 PMC9828552

[CR39] Matcham G, Bhatia M, Lang W, Lewis C, Nelson R, Wang A, Wu W (1999) Enzyme and reaction engineering in biocatalysis: Synthesis of (S)-Methoxyisopropylamine (= (S)-1-Methoxypropan-2-amine). Chimia 53(12):584. 10.2533/chimia.1999.584

[CR40] Mayol O, David S, Darii E, Debard A, Mariage A, Pellouin V, Petit JL, Salanoubat M, de Berardinis V, Zaparucha A, Vergne-Vaxelaire C (2016) Asymmetric reductive amination by a wild-type amine dehydrogenase from the thermophilic bacteria *Petrotoga mobilis*. Catal Sci Technol 6(20):7421–7428. 10.1039/C6CY01625A

[CR41] Mayol O, Bastard K, Beloti L, Frese A, Turkenburg JP, Petit JL, Mariage A, Debard A, Pellouin V, Perret A, de Berardinis V, Zaparucha A, Grogan G, Vergne-Vaxelaire C (2019) A family of native amine dehydrogenases for the asymmetric reductive amination of ketones. Nat Catal 2(4):324–333. 10.1038/s41929-019-0249-z

[CR42] Meng Z, Cui J, Li R, Sun W, Bao X, Wang J, Zhou Z, Zhu W, Chen X (2022) Systematic evaluation of chiral pesticides at the enantiomeric level: a new strategy for the development of highly effective and less harmful pesticides. Sci Total Environ 846:157294. 10.1016/j.scitotenv.2022.157294 35839878 10.1016/j.scitotenv.2022.157294

[CR43] Mhadhbi L, Beiras R (2016) Retraction note: Acute toxicity of seven selected pesticides (alachlor, atrazine, dieldrin, diuron, pirimiphos-methyl, chlorpyrifos, diazinon) to the marine fish (turbot, Psetta maxima). Water Air Soil Pollut 227(8):275. 10.1007/s11270-016-2980-2

[CR44] Midelfort KS, Kumar R, Han S, Karmilowicz MJ, McConnell K, Gehlhaar DK, Mistry A, Chang JS, Anderson M, Villalobos A, Minshull J, Govindarajan S, Wong JW (2013) Redesigning and characterizing the substrate specificity and activity of Vibrio fluvialis aminotransferase for the synthesis of imagabalin. Protein Eng des Sel 26(1):25–33. 10.1093/protein/gzs065 23012440 10.1093/protein/gzs065

[CR45] Mikula P, Modra H, Nemethova D, Groch L, Svobodova Z (2008) Effects of subchronic exposure to LASSO MTX® (alachlor 42% W/V) on hematological indices and histology of the common carp, Cyprinus carpio L. Bull Environ Contam Toxicol 81(5):475–479. 10.1007/s00128-008-9500-z 18654729 10.1007/s00128-008-9500-z

[CR46] Moser H, Rihs G, Sauter HP, Böhner B (1983) Atropisomerism, chiral centre and activity of metolachlor. In: Doyle P, Fujita T (eds) Pesticide chem. Elsevier, Amsterdam, pp 315–320

[CR47] Mutti FG, Knaus T, Scrutton NS, Breuer M, Turner NJ (2015) Conversion of alcohols to enantiopure amines through dual-enzyme hydrogen-borrowing cascades. Science 349(6255):1525–1529. 10.1126/science.aac9283 26404833 10.1126/science.aac9283 PMC4883652

[CR48] Nugent WA, Rajanbabu TV, Burk MJ (1993) Beyond nature’s chiral pool: Enantioselective catalysis in industry. Science 259(5094):479–483. 10.1126/science.259.5094.479 17734166 10.1126/science.259.5094.479

[CR49] Rehn G, Ayres B, Adlercreutz P, Grey C (2016) An improved process for biocatalytic asymmetric amine synthesis by in situ product removal using a supported liquid membrane. J Mol Catal B Enzym 123:1–7. 10.1016/j.molcatb.2015.10.010

[CR50] Rusek M (1991) Effect of promoters on Pt/SiO2 catalysts for the N-alkylation of sterically hindered anilines in the vapor phase. J Mol Catal 59:359–365. 10.1016/S0167-2991(08)61142-8

[CR51] Savile CK, Janey JM, Mundorff EC, Moore JC, Tam S, Jarvis WR, Colbeck JC, Krebber A, Fleitz FJ, Brands J, Devine PN, Huisman GW, Hughes GJ (2010) Biocatalytic asymmetric synthesis of chiral amines from ketones applied to sitagliptin manufacture. Science 329(5989):305–309. 10.1126/science.1188934 20558668 10.1126/science.1188934

[CR52] Schrittwieser JH, Velikogne S, Kroutil W (2015) Biocatalytic imine reduction and reductive amination of ketones. Adv Synth Catal. 10.1002/adsc.201500213

[CR53] Seckinger K, Kuhnen F, Milzner K (1983) 5-Membered hetero: Aromatic chloro: Acetamide derivs.—selective herbicides effective against both mono-cotyledons and dicotyledons. DE Patent 3,303,388.

[CR54] Seckinger K, Kuhnen F, Milzner K (1987) Herbicidal N-thienyl-chloroacetamides. US Patent 4,666,502.

[CR55] Seckinger K, Chollet R, Blarer S, Vettiger T (1995) Optical isomer of dimethenamid. US Patent 5,457,085.

[CR56] Su SQ (2002) Review of amide herbicides. Agrochem 11:1–5. 10.16820/j.cnki.1006-0413.2002.11.001

[CR57] Sun K (2013) Market and outlook of the top ten herbicides in the world. Agrochem 52(5):317–322. 10.16820/j.cnki.1006-0413.2013.05.002

[CR58] Tseliou V, Knaus T, Masman MF, Corrado ML, Mutti FG (2019) Generation of amine dehydrogenases with increased catalytic performance and substrate scope from ε-deaminating L-Lysine dehydrogenase. Nat Commun 10(1):3717. 10.1038/s41467-019-11509-x 31420547 10.1038/s41467-019-11509-x PMC6697735

[CR59] Vashistha VK, Sethi S, Mittal A, Das DK, Pullabhotla RVSR, Bala R, Yadav S (2024) Stereoselective analysis of chiral pesticides: a review. Environ Monit Assess 196(2):153. 10.1007/s10661-024-12310-0 38225517 10.1007/s10661-024-12310-0

[CR60] Wang LZ, Sun K, Zhang MH (2014) A review of synthetic methods of dimethenamid-P. Agrochem 53(4):307–309. 10.16820/j.cnki.1006-0413.2014.04.024

[CR61] Wang H, Zheng YC, Chen FF, Xu JH, Yu HL (2020) Enantioselective bioamination of aromatic alkanes using ammonia: a multienzymatic cascade approach. ChemCatChem 12(7):2077–2082. 10.1002/cctc.201902253

[CR62] Wang Y, Feng J, Dong W, Chen X, Yao P, Wu Q, Zhu D (2021) Improving catalytic activity and reversing enantio-specificity of ω-transaminase by semi-rational engineering en route to chiral bulky β-amino esters. ChemCatChem 13(15):3396–3400. 10.1002/cctc.202100503

[CR63] Wu F, Wang Z, Li X, Wang X (2023) Amide herbicides: Analysis of their environmental fate, combined plant-microorganism soil remediation scheme, and risk prevention and control strategies for sensitive populations. J Hazard Mater 15(460):132452. 10.1016/j.jhazmat.2023.132452 10.1016/j.jhazmat.2023.132452 37683346

[CR64] Wu W, Bhatia MB, Lewis CM, Lang W, Wang L, Macam GW (2001) Improved enzymatic synthesis of chiral amines. CN 1292828A.

[CR65] Xiang C, Ao YF, Höhne M, Bornscheuer UT (2022) Shifting the pH optima of (R)-selective transaminases by protein engineering. Int J Mol Sci 23(23):15347. 10.3390/ijms232315347 36499674 10.3390/ijms232315347 PMC9736275

[CR66] Xue YP, Cao CH, Zheng YG (2018) Enzymatic asymmetric synthesis of chiral amino acids. Chem Soc Rev 47(4):1516–1561. 10.1039/C7CS00253J 29362736 10.1039/c7cs00253j

[CR67] Yang LR, Zhang T, Zhou HS, Zhang HY, Wu JP (2022) Application of transaminase and its mutants in the preparation of (S)-1-methoxy-2-propylamine. CN 114134126A.

[CR68] Ye LJ, Toh HH, Yang Y, Adams JP, Snajdrova R, Li Z (2015) Engineering of amine dehydrogenase for asymmetric reductive amination of ketone by evolving *Rhodococcus* phenylalanine dehydrogenase. ACS Catal 5(2):1119–1122. 10.1021/cs501906r

[CR69] Yuan M, Kummer MJ, Milton RD, Quah T, Minteer SD (2019) Efficient NADH regeneration by a redox polymer-immobilized enzymatic system. ACS Catal 9(6):5486–5495. 10.1021/acscatal.9b00513

[CR70] Zhang YB (2011a) Market and products and development trend of amide herbicides. Mod Agrochem 10(1):41–43. 10.3969/j.issn.1671-5284.2011.01.012

[CR71] Zhang HB (2011b) Study on synthesis of S-metolachlor. Pestic Sci Adm 32(11):26–29

[CR72] Zhang X, Yang RL, Liu CH, Sun YM, Lei HT (2015) Progress in toxicity of chiral herbicide metolachlor. Nat Sci Ed 43(1):152–158. 10.13207/j.cnki.jnwafu.2015.01.015

[CR73] Zhang WG, Zhang SL, Guo D, Zhao L, Yu LJ, Zhang H, He YJ (2019) Great concern for chiral pharmaceuticals from the thalidomide tragedy. Univ Chem 34(9):1–12. 10.3866/PKU.DXHX201904021

[CR74] Zhao J, Hu XX, Liu Q, Bai LY, Jin CZ, Li JB (2015) Sensitivities of six different rice varieties to metolachlor. Plant Prot 41(2):176–180. 10.3969/j.issn.0529-1542.2015.02.034

[CR75] Zheng LY, Zheng L, Zhang S, Wang F, Gao G, Cao S (2006) Chemoenzymatic synthesis of the chiral herbicide: S-metolachlor. Can J Chem 84(8):1058–1063. 10.1139/v06-129

[CR76] Zheng LY (2005) Study on lipase-catalyzed resolution of N-substituted phenyl α-alanine. Dissertation, Jilin University.

[CR77] Zhou J, Liang SL, Xiang TT, Rong Y, Lü DZ (2021) Research progress on chiral pesticides. Acta Agric Jiangxi 33(7):75–80. 10.19386/j.cnki.jxnyxb.2021.07.012

[CR78] Zhu FY, Huang MY, Zheng K, Zhang XJ, Cai X, Huang LG, Liu ZQ, Zheng YG (2023) Designing a novel (R)-ω-transaminase for asymmetric synthesis of sitagliptin intermediate via motif swapping and semi-rational design. Int J Biol Macromol 253:127348. 10.1016/j.ijbiomac.2023.127348 37820904 10.1016/j.ijbiomac.2023.127348

[CR79] Zhu HJ, Li YF, Chen W, Yu GQ, Du G, Lü LZ, Chu QY (2009) A novel method for synthesizing (S)-metolachlor. CN 101367746A,

